# Association of macrophage inhibitory cytokine-1 with nutritional status, body composition and bone mineral density in patients with anorexia nervosa: the influence of partial realimentation

**DOI:** 10.1186/1743-7075-7-34

**Published:** 2010-04-23

**Authors:** Ivana Dostálová, Petra Kaválková, Hana Papežová, Daniela Domluvilová, Vít Zikán, Martin Haluzík

**Affiliations:** 1 3rd Department of Medicine, 1st Faculty of Medicine, Charles University and General University Hospital, U nemocnice 1, 128 08 Prague 2, Czech Republic; 2 Department of Psychiatry, 1st Faculty of Medicine, Charles University and General University Hospital, Ke Karlovu 11, 121 08 Prague 2, Czech Republic

## Abstract

**Background:**

Macrophage inhibitory cytokine-1 (MIC-1) is a key inducer of cancer-related anorexia and weight loss. However, its possible role in the etiopathogenesis of nutritional disorders of other etiology such as anorexia nervosa (AN) is currently unknown.

**Methods:**

We measured fasting serum concentrations of MIC-1 in patients with AN before and after 2-month nutritional treatment and explored its relationship with nutritional status, metabolic and biochemical parameters. Sixteen previously untreated women with AN and twenty-five normal-weight age-matched control women participated in the study. We measured serum concentrations of MIC-1 and leptin by ELISA, free fatty acids by enzymatic colorimetric assay, and biochemical parameters by standard laboratory methods; determined resting energy expenditure by indirect calorimetry; and assessed bone mineral density and body fat content by dual-energy X-ray absorptiometry. ANOVA, unpaired t-test or Mann-Whitney test were used for groups comparison as appropriate. The comparisons of serum MIC-1 levels and other studied parameters in patients with AN before and after partial realimentation were assessed by paired t-test or Wilcoxon Signed Rank Test as appropriate.

**Results:**

At baseline, fasting serum MIC-1 concentrations were significantly higher in patients with AN relative to controls. Partial realimentation significantly reduced serum MIC-1 concentrations in patients with AN but it still remained significantly higher compared to control group. In AN group, serum MIC-1 was inversely related to Buzby nutritional risk index, serum insulin-like growth factor-1, serum glucose, serum total protein, serum albumin, and lumbar bone mineral density and it significantly positively correlated with the duration of AN and age.

**Conclusions:**

MIC-1 concentrations in AN patients are significantly higher relative to healthy women. Partial realimentation significantly decreased MIC-1 concentration in AN group. Clinical significance of these findings needs to be further clarified.

## Background

Anorexia Nervosa (AN) represents a bordering example of psychosomatic-based malnutrition induced by chronically decreased food intake caused by inappropriate fear of obesity and distorted body image [1-4]. The exact etiopathogenesis of AN is still not completely understood. Although alterations in circulating levels of many appetite-regulating hormones, including leptin and ghrelin, in concert with many abnormalities in the metabolism of adipose and other tissues have been previously documented in patients with AN [5-13] most of these changes appear rather secondary to compensate for chronically decreased food intake and malnutrition. None of these factors can explain the anorectic behavior and prolonged tendency towards weight loss of these patients. In fact, the factors contributing to the chronic course of AN are mostly unknown.

Macrophage inhibitory cytokine-1 (MIC-1) is a member of the transforming growth factor-β superfamily [14]. It is strongly expressed in activated macrophages (14), adipocytes [15], placenta and prostate, and to lesser extent in the liver, kidney and brain [16,17]. Significant amounts of MIC-1 are released into the circulation [14,18,19] suggesting that it can also act as an endocrine factor [20].

MIC-1 is involved in the control of multiple cellular processes, being markedly increased in patients with chronic inflammatory states such as rheumatoid arthritis [21] and overexpressed by many types of malignant tumors [22-26]. MIC-1 was also identified as a predictor of cardiovascular events in a cohort of previously healthy women [27], as a new marker predicting death and heart failure in post-myocardial infarction patients [28], and as an early mediator of the injury response in kidney and lung [29].

Recently, a key role of increased MIC-1 concentrations in the induction of cancer-related anorexia and weight loss in prostate tumor-bearing mice and in humans with advanced prostate cancer was described [30]. In the brain, MIC-1 decreased food intake through modulation of neuropeptide Y and proopiomelanocortin levels in nucleus arcuatus [30]. Administration of MIC-1 antibody to tumor-bearing mice rapidly reversed the weight loss, whereas MIC-1 administration to normal mice or its transgenic overexpression induced hypophagia and decreased body weight [30]. Additionally, MIC-1 gene expression was recently identified in human subcutaneous and visceral fat and it was inversely related to body mass index and body fat mass [15].

To our best knowledge MIC-1 has not been previously studied in patients with AN. Chronic malnutrition in patients with AN shares some common hormonal and metabolic features with cancer anorexia [31]. Therefore, we hypothesized that elevated serum levels of MIC-1 may contribute to reduced appetite, weight loss or possibly other metabolic and nutritional disturbances in patients with AN. To this end, we measured fasting serum levels of MIC-1 in patients with AN and in healthy normal-weight women and studied its relationship to clinical characteristics, body composition, bone mineral density and various hormonal and metabolic parameters. Furthermore, we studied the effect of partial realimentation of patients with AN on serum MIC-1 concentrations and its relationship to above described clinical characteristics.

## Methods

### Study subjects

Sixteen previously untreated female patients with AN (age: 24.9 ± 1.34 years; body mass index (BMI): 15.7 ± 0.30 kg/m^2^; body fat content (DEXA): 13.9 ± 1.44%) and twenty-five age- and sex-matched healthy controls (age: 22.9 ± 0.50 years; BMI: 21.8 ± 0.38 kg/m^2^; body fat content (DEXA): 26.4 ± 1.79%) were included in the study. The diagnosis of eating disorder was based on Diagnostic Statistical Manual IV diagnostic system (DSM-IV). A clinical evaluation of the patients was performed by an experienced psychiatrist. The Structured Clinical Interview MINI 5.0 was used for diagnostic assessment of the patients. None of the studied subjects suffered from diabetes mellitus, thyroid disorder and/or acute infectious disease. None of the studied subjects had malignant tumor. All women included in the study had no allergies. Healthy control women had been free of any medication for at least three months prior to the study and had no history of obesity or malnutrition, hypertension, gastrointestinal disease, eating disorder or other psychiatric disorder. Blood tests confirmed normal blood count, liver and renal functions. All healthy women had regular menstrual cycle. The baseline examination of the patients was performed before the start of any treatment. At that time all patients were free of any medication affecting food intake and/or appetite.

Written informed consent was provided by all participants before being enrolled in the study. The study was approved by the Human Ethical Review Committee, First Faculty of Medicine and General University Hospital, Prague, Czech Republic, and was performed in accordance with the guidelines proposed in the Declaration of Helsinki.

### Anthropometric examination and blood sampling

All patients with AN were examined twice; at a basal state before the beginning of any treatment and after two months of partial realimentation while normal-weight healthy women were examined only once. All subjects were measured and weighted and BMI was calculated. Body fat content was estimated by bioimpedance analysis (Bodystat 1500, Bodystat Ltd., UK) and by dual energy X-ray absorptiometry (DEXA). Bone mineral density was also estimated by DEXA on QDR 4500A bone densitometer (Hologic, Waltham, MA, USA). Resting energy expenditure was determined by indirect calorimetry (Vmax ENCORE Viasys^TH ^HEALTHCARE, SensorMedics BV, Netherlands). Blood samples for MIC-1, and biochemical parameters measurements were withdrawn between 0700 and 0800 h after 12 h of overnight fasting. Serum was separated by centrifugation and stored at -80°C until being assayed.

Nutritional risk index by Buzby (NRI) was calculated according the following formula: NRI = 1.519 + albumin (g/l) + 0.417 * actual body weight/ideal body weight * 100. Ideal body weight was calculated as (0.593*body height) - 38.6. Strong malnutrition is defined by NRI < 83.5 [32].

### Hormonal and biochemical assays

Serum MIC-1 concentrations were measured by a commercial ELISA kit (BioVendor, Brno, Czech Republic). Sensitivity was 10 pg/ml and the intra- and inter-assay variability was 3.7% and < 10%. Serum leptin concentrations were measured by commercial ELISA kit (BioVendor, Brno, Czech Republic). Sensitivity was 0.12 ng/ml and the intra- and inter-assay variability was 1.7 and 8.0%, respectively. Serum concentrations of insulin-like growth factor-1 (IGF-1) were measured by commercial IRMA kit (Immunotech, Prague, Czech Republic). Sensitivity was 2 ng/ml, and the intra- and inter-assay variability was 6.3 and 6.8%, respectively. Serum levels of free fatty acids were measured by enzymatic colorimetric assay (Roche, Basel, Switzerland). Serum biochemical parameters (glucose, total and HDL-cholesterol, triglycerides and albumin) were measured by standard laboratory methods on Modular SWA analyzer (Roche Diagnostics, GmbH, Mannheim, Germany), the value of LDL-cholesterol was calculated by Friedewald formula.

### Statistical analysis

The statistical analysis was performed on SigmaStat software (Jandel Scientific, San Rafael, CA). Results are expressed as mean ± S.E.M. ANOVA, unpaired t-test or Mann-Whitney test were used for groups comparison as appropriate. The comparisons of serum MIC-1 levels and other studied parameters in patients with AN before and after partial realimentation were assessed by paired t-test or Wilcoxon Signed Rank Test as appropriate. The univariate correlations between MIC-1 and other parameters were estimated by Spearman Correlation Test. Multiple regression analysis was used to show the independent relationship of other parameters with MIC-1. A p value < 0.05 denoted statistical significance.

## Results

### Anthropometric, hormonal and biochemical characteristics of the study subjects

The study groups were age-matched. Patients with AN had severely decreased BMI and body fat content (Table 1). At baseline, patients with AN had significantly decreased lean body mass and daily resting energy expenditure as compared with control group. Fasting serum concentrations of leptin, glucose, free fatty acids, and total serum protein were significantly reduced in AN group as compared with C group. Fasting LDL cholesterol levels in untreated AN patients did not significantly differ from C group.

**Table 1:** Characteristics of the study groups.

	Controls (n = 25)	AN pre-treatment (n = 16)	AN post-treatment (n = 16)
Age (years)	22.9 ± 0.50	24.9 ± 1.34	24.9 ± 1.34
Body mass index (kg/m^2^)	21.8 ± 0.38	15.7 ± 0.30*	17.5 ± 0.23*^+^
Body fat content (bodystat) (%)	21.9 ± 1.21	9.3 ± 1.65*	12.8 ± 1.38*^+^
Body fat content (DEXA) (%)	26.4 ± 1.79	13.9 ± 1.44*	18.0 ± 1.38*^+^
Lean body mass (kg)	52.5 ± 2.03	38.9 ± 1.3*	41.3 ± 0.96*
Resting energy expenditure (kcal/day)	1400.1 ± 35.92	1060.0 ± 40.25*	1176.3 ± 25.05*^+^
Fasting glucose (mmol/l)	4.4 ± 0.07	3.9 ± 0.09*	4.2 ± 0.08^+^
Total cholesterol (mmol/l)	4.2 ± 0.14	4.6 ± 0.26	5.2 ± 0.27*^+^
HDL-cholesterol (mmol/l)	1.6 ± 0.08	1.5 ± 0.10	1.6 ± 0.08
LDL-cholesterol (mmol/l)	2.1 ± 0.09	2.4 ± 0.18	3.0 ± 0.21*^+^
Free fatty acids (mmol/l)	0.64 ± 0.06	0.16 ± 0.02*	Not measured
Total serum protein (g/l)	79.1 ± 1.49	68.7 ± 2.01*	75.5 ± 1.04^+^
Albumin (g/l)	46.4 ± 1.22	43.8 ± 1.12	47.6 ± 0.72^+^
Total bone mineral density (g/cm^2^)	1.09 ± 0.02	1.04 ± 0.02*	Not measured
Lumbar (L1-L4) bone mineral density (g/cm^2^)	1.03 ± 0.03	0.86 ± 0.03*	Not measured
Proximal femur bone mineral density (g/cm^2^)	0.98 ± 0.02	0.78 ± 0.04*	Not measured
**IGF-1 (ng/ml)**	**NA**	**156 **± 18	**NA**

**MIC-1 (pg/ml)**	**1051 ± 83**	1872 ± 200*	1530 ± 169*^+^

Leptin (ng/ml)	11.8 ± 1.78	0.97 ± 0.23*	2.6 ± 0.66*^+^

Anthropometric, hormonal and biochemical characteristics of the control group of normal-weight healthy women and patients with anorexia nervosa both before (AN pre-treatment) and after (AN post-treatment) treatment. Values are means ± SEM. Statistical significance is from unpaired t-test or Mann-Whitney test (AN vs. controls) and from paired t-test or Wilcoxon Signed Rank Test (post- vs. pre-treatment values in AN) as appropriate. *p < 0.05 vs. controls; ^+^p < 0.05 vs. pre-treatment values. NA - not available

Fasting serum HDL-cholesterol, total cholesterol and albumin concentrations did not significantly differ between the groups. At baseline, lumbar, proximal femur and total bone mineral density were significantly reduced in AN group relative to C group (Table 1).

The influence of partial realimentation on anthropometric, hormonal, and biochemical characteristics of patients with AN is summarized in Table 1. Patients with AN significantly gained weight and body fat content during realimentation. Fasting serum leptin, total serum cholesterol, glucose, total protein, albumin, LDL-cholesterol concentrations and daily resting energy expenditure significantly increased in patients with AN after partial refeeding as compared with pre-treatment values. Post-treatment fasting serum HDL-cholesterol concentrations were not significantly different as compared with its pre-treatment levels in AN. Post-treatment concentrations of total cholesterol and LDL-cholesterol were significantly increased in patients with AN as compared with controls (Table 1).

### Serum levels of MIC-1 in AN and normal-weight women and its changes after two-month realimentation

**Figure 1:**
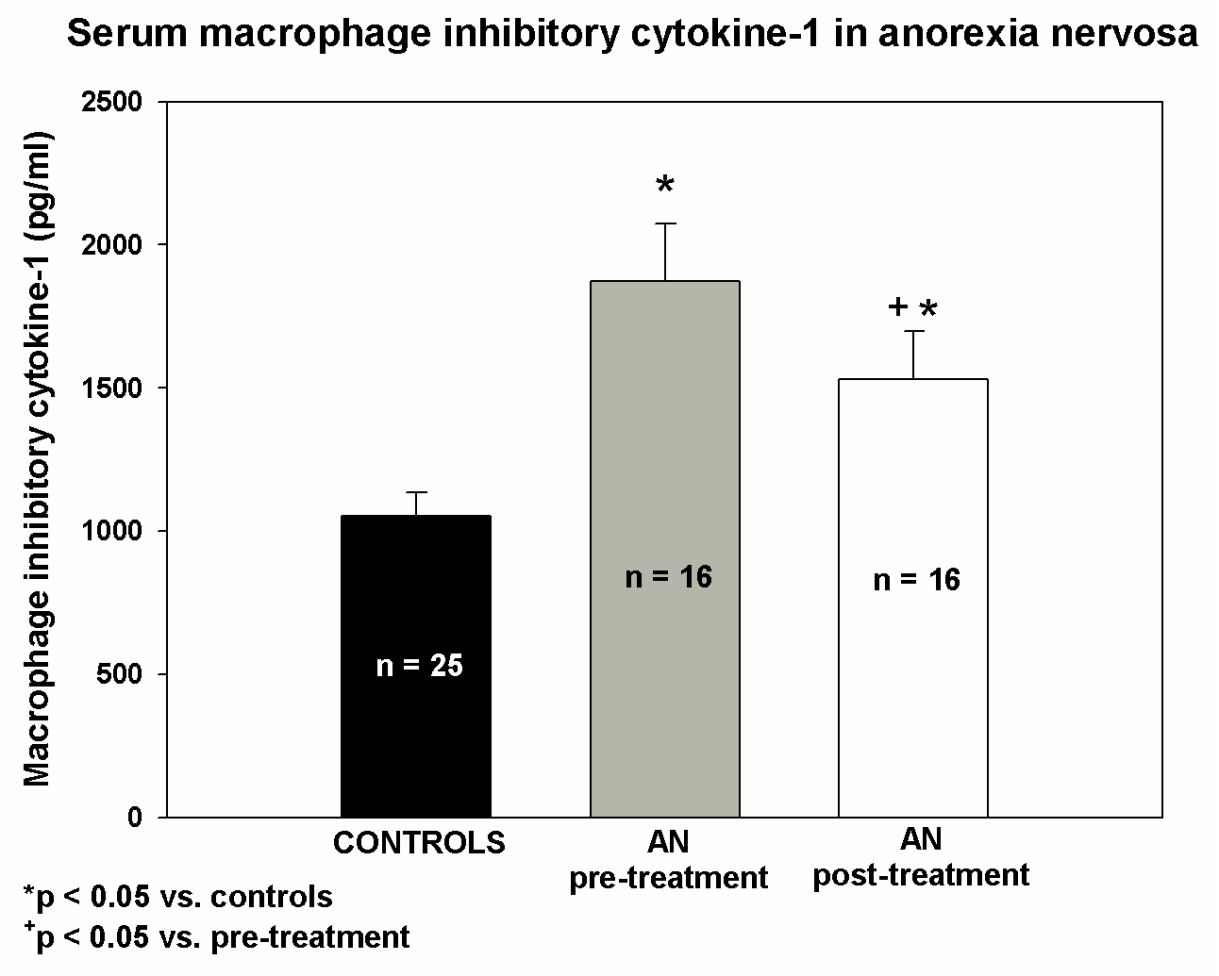
**Serum concentrations of macrophage inhibitory cytokine-1 in anorexia nervosa**. Serum concentrations of macrophage inhibitory cytokine-1 (MIC-1; pg/ml) in normal-weight healthy control women (C; n = 25), patients with anorexia nervosa before the beginning of any treatment (AN pre-treatment; n = 16) and patients with anorexia nervosa after two months of refeeding (AN post-treatment; n = 16). *p < 0.05 vs. controls (Mann-Whitney test), ^+^p < 0.05 vs. pre-treatment values (Wilcoxon Signed Rank Test).

At baseline, fasting serum MIC-1 concentrations were significantly higher in patients with AN relative to control group of healthy normal-weight women (Figure 1). Partial realimentation significantly reduced serum MIC-1 concentrations in patients with AN but it still remained significantly higher compared to controls (Figure 1).

### Relationship of MIC-1 with other studied parameters

The relationship of serum MIC-1 levels with other parameters was assessed in a combined population of both groups and separately in patients with AN (Tables 2 and 3). In a combined population (Table 2), serum pre-treatment MIC-1 concentrations were correlated with other pre-treatment parameters by Spearman correlation test. The correlations were normalized for BMI. In a combined group of AN and controls pre-treatment MIC-1 positively correlated with age and serum total cholesterol, and was inversely related to BMI, lean body mass, serum glucose, serum albumin, serum leptin, lumbar bone mineral density and resting energy expenditure (Table 2).

**Table 2:** Relationships of MIC-1 with other parameters in combined population.

AN + C (n = 41)		BMI	LBM	Glucose	Cholesterol	Albumin	BMD lumbar	Leptin	REE	age
MIC1	r	-0.52	-0.59	-0.36	0.47	-0.49	-0.53	-0.43	-0.64	-0.57
	p	0.000	0.001	0.02	0.002	0.008	0.003	0.04	0.000	0.000

Statistically significant relationships of pre-treatment concentrations of macrophage inhibitory cytokine-1 (MIC-1) with anthropometric, hormonal and biochemical parameters calculated in a combined population of normal-weight healthy women and untreated patients with anorexia nervosa (n = 41). Statistical significance is from Spearman Correlation Test. Correlations were controlled for body mass index (BMI). AN - anorexia nervosa, C - control group, LBM - lean body mass, BMD - bone mineral density, REE - resting energy expenditure

**Table 3:** Relationships of MIC-1 with other parameters in patients with anorexia nervosa.

AN (n = 16)		NRI-2	IGF-1	GLU-2	TP-2	albumin-2	BMD lumbar	duration of illness	age
Pre-treatment MIC1	r	-0.60	- 0.65	NS	-0.65	-0.55	NS	NS	0.53
	p	0.01	0.02		0.007	0.03			0.03
Post-treatment MIC1	r	-0.68	- 0.60	-0.62	-0.52	-0.70	-0.55	0.53	0.51
	p	0.004	0.04	0.01	0.04	0.002	0.03	0.03	0.04

Relationships of pre-treatment and post-treatment concentrations of macrophage inhibitory cytokine-1 (MIC-1) with anthropometric, hormonal and biochemical parameters calculated separately in a group of anorexia nervosa patients (n = 16). Statistical significance is from Spearman Correlation Test. Correlations were controlled for body mass index (BMI). AN - anorexia nervosa, C - control group, LBM - lean body mass, BMD - bone mineral density, NRI-2 - post-treatment nutritional risk index (Buzby), IGF-1, insulin-like growth factor, GLU-2 - post-treatment glucose, TP-2, post-treatment total serum protein

In patients with AN, univariate correlations were calculated by Spearman correlation test for pre-treatment MIC-1 levels with other pre- and post-treatment parameters (Table 3) and post-treatment MIC-1 levels with other pre- and post-treatment parameters (Table 3), respectively. Both pre- and post-treatment serum MIC-1 levels were inversely related to post-treatment Buzby nutritional risk index, pre-treatment serum insulin-like growth factor concentrations, post-treatment total protein and post-treatment albumin levels. Both pre- and post-treatment MIC-1 levels positively correlated with age. Post-treatment serum MIC-1 levels significantly inversely correlated with post-treatment serum glucose and pre-treatment lumbar bone mineral density and were significantly positively associated with the duration of illness in patients with AN (Table 3). In both groups, we failed to find any significant relationship of MIC-1 with body fat content, serum thyroid hormones (fT3, fT4, TSH), serum free fatty acids, and liver enzymes (aspartate aminotransferase, alanine aminotransferase).

In patients with AN, delta MIC-1 during treatment significantly inversely correlated with delta LBM (r = - 0.70, p = 0.002) during treatment.

Multiple regression analysis was performed in combined population with pre-treatment MIC-1 levels as dependent and pre-treatment BMI, lean body mass, glucose, cholesterol, albumin, lumbar bone mineral density, leptin, resting energy expenditure and age as independent variables. None of the factors included was identified as statistically significant independent predictor of pre-treatment MIC-1 concentrations.

Multiple regression analyses were also performed separately in anorexia nervosa group with pre-treatment MIC-1 and post-treatment MIC-1 levels as dependent parameters, respectively. First multiple regression analysis was performed with pre-treatment MIC-1 levels as dependent and pre- and post-treatment Buzby nutritional index, pre-treatment IGF-1, lumbar bone mineral density, age, duration of illness and post-treatment glucose, total protein and albumin as independent variables. Similarly as in the combined group of AN and control subjects none of the factors included was identified as statistically significant independent predictor of pre-treatment MIC-1 concentrations.

Another multiple regression analysis was performed for post-treatment MIC-levels as dependent and pre- and post-treatment Buzby nutritional index, pre-treatment IGF-1, lumbar bone mineral density, age, duration of illness and post-treatment glucose, total protein and albumin as independent variables. Similarly as in previous cases none of the factors included was identified as statistically significant independent predictor of pre-treatment MIC-1 concentrations.

Finally, to assess a possible role of MIC-1 levels in the regulation bone mineral density two multiple regression analyses were performed. In the first one, pre-treatment bone mineral density was set as dependent variable and age, post-treatment MIC-1, post-treatment blood glucose, post-treatment BMI, post-treatment IGF-1, post-treatment albumin and post-treatment Buzby nutritional index as independent variables. In the second one, pre-treatment bone mineral density was set as dependent variable and age, pre-treatment MIC-1, pre-treatment blood glucose, pre-treatment BMI, pre-treatment IGF-1, pre-treatment albumin and pre-treatment Buzby nutritional index as independent variables. None of the factors included was identified as statistically significant independent predictor of pre-treatment bone mineral density.

## Discussion

The most important finding of the present study is that chronically malnourished patients with AN have significantly increased serum MIC-1 concentrations as compared with healthy normal-weight women. Partial realimentation improved their nutritional status and significantly reduced serum MIC-1 concentrations in AN. Furthermore, serum MIC-1 significantly inversely correlated with Buzby nutritional risk index, serum insulin-like growth factor-1, serum glucose, serum total protein, serum albumin, and bone mineral density in women with AN. Post-treatment serum MIC-1 concentrations significantly positively correlated with the duration of AN.

The overexpression and elevated serum levels of MIC-1 have been previously documented in rheumatoid arthritis and in numerous types of cancer [22-26]. Although serum MIC-1 levels have been shown to predict the diagnosis and metastatic progression of various cancers [22,25], its exact mode of action and major effects in humans are still only partially understood. In fact, there is only one study that clearly described a direct role of MIC-1 as an inducer of cancer-related anorexia and weight loss in mice [30]. Here we show for the first time that severely malnourished patients with AN, similarly to cachectic cancer patients, exhibit elevated serum MIC-1 levels. However, the nature of our study does not allow us to determine whether elevated MIC-1 levels play a role in chronic metabolic complications of anorexia nervosa or whether they are rather a consequence of chronic malnutrition of these patients.

Another important finding of our study is the fact that elevated serum MIC-1 concentrations in patients with AN were significantly reduced by partial realimentation; albeit they still remained significantly higher than in healthy women. Reduction of serum MIC-1 during realimentation in AN may due to increased lean body mass together with partial normalization of some of metabolic parameters (total serum protein, albumin, glucose) during realimentation. One can speculate that decreased MIC-1 concentrations during nutritional treatment can also be a part of useful adaptive mechanism to increase the feeding drive in the state of refeeding after long-term starvation [30]. Alternatively, changes of circulating MIC-1 levels after partial realimentation may suggest that MIC-1 is a marker of response to refeeding in anorexia nervosa patients.

The duration of the illness significantly influences the degree and severity of long-term nutritional, metabolic and endocrine abnormalities in AN [33-36]. It was previously postulated, that in bulimic patients, circulating leptin was inversely related with the duration of the illness and the frequency of binging/vomiting [37]. However, to our best knowledge, the biological markers of the chronicity of AN are mostly unknown. Here we showed that serum MIC-1 concentrations in patients with AN positively correlated with the duration of the illness and age. Higher age of patients with anorexia nervosa commonly suggests longer duration of illness increasing the likelihood of chronic complications. Collectively, our results suggest the possible relationship of MIC-1 concentrations with the duration of anorexia nervosa and possibly also with its chronic course.

Osteopenia/osteoporosis is one of the most frequent consequences of AN that is significantly influenced by the duration of the disease [38]. Monitoring of decreased bone mineral density in AN by DEXA can be used as an effective screening tool and should be probably offered routinely [39]. It is thus very important to identify predictors of decreased BMD in AN to pinpoint the AN patients with higher risk of osteoporosis that should undergo DEXA examination. Previous studies postulated that BMI [34], duration of the illness, presence of amenorrhea [40] and peptide YY [41] were strongly associated with decreased BMD in patients with AN. Here we found in univariate correlation analysis that post-treatment circulating MIC-1 levels were inversely related to lumbar BMD. In multiple regression analysis with BMD as independent variable after inclusion of body mass index among independent parameters relationship between both pre- and post-treatment circulating MIC-1 levels and lumbar BMD was not statistically significant anymore arguing rather against direct independent role of MIC-1 in the regulation of bone metabolism in AN patients. It has to be noted that the negative results of multiple regression analysis with respect to association of BMD and circulating MIC-1 levels could have been also affected by relatively low numbers of patients in our study.

Our results are in agreement with proposed role of MIC-1 in wasting syndrome such as cancer cachexia and AN. In contrast to cancer cachexia where MIC-1 is predominantly produced by tumors, the origin of circulating MIC-1 in patients with AN is unclear and should be further investigated. MIC-1 is significantly increased under inflammatory conditions [21] and is extensively produced by macrophages [14] and also by adipocytes [15]. Taken together, these findings suggest that adipose tissue may be in general one of the sources of MIC-1. However, the major contribution of fat to the production of MIC-1 in anorectic patients appears questionable in the context of decreased total fat mass in these patients. Moreover, MIC-1 concentrations in AN decreased after partial refeeding and weight gain when body fat content increases and we failed to find any relationship between body fat content and serum MIC-1 levels in our cohort of patients. In addition, we have recently described increased levels of circulating MIC-1 also in obese and type 2 diabetic women [42]. The fact that circulating levels of MIC-1 are increased in cachectic cancer and AN patients as well as in patients with obesity despite opposite changes in body weight and body fat content argues rather against a direct role for body weight and body fat content *per se *in the regulation of circulating MIC-1 levels.

Previous studies indicated a possible involvement of oxidative stress in the induction of MIC-1 synthesis in humans [15]. The presence of increased oxidative stress [43-45] could potentially explain why both malnourished and obese patients display increased MIC-1 levels. We suggest that both extreme cachexia in patients with cancer and AN and morbid obesity may result in nutritional stress and subsequently oxidative stress in adipocytes and other tissues which may in turn stimulate MIC-1 production. The design and other limitations of our study do not allow us to directly test this hypothesis. Nevertheless, we have previously demonstrated that local changes in mRNA expression of some of proinflammatory cytokines in subcutaneous adipose tissue of malnourished patients with AN share some similarities with changes in patients with obesity suggesting that local inflammatory response is present in adipose tissue of both cachectic and obese patients [46,47]. Another possible site of MIC-1 production may be the liver [48].

## Conclusions

We demonstrated that AN is accompanied by significantly elevated serum MIC-1 levels that are reduced by partial realimentation. Clinical significance of these findings and possible role of MIC-1 in the etiopathogenesis of anorexia nervosa and its chronic complications needs to be further clarified.

## Competing interests

The authors declare that they have no competing interests.

## Authors' contributions

Authors' contribution to the work: ID and MH - conception, design, and conduct of the study, interpretation and statistical evaluation of the data; PK - examination of the study subjects and data assembly; HP and DD selection and clinical evaluation of the patients (psychiatrists); VZ DEXA measurements and clinical evaluation of the study subjects (physician). All authors read and approved the final manuscript.
